# Supplementary material for: Immunogenic Cell Death Associated Molecular Patterns and the Dual Role of IL17RA in Interstitial Cystitis/Bladder Pain Syndrome
Source: Biomolecules. 2023 Feb 23;13(3):421. doi: 10.3390/biom13030421 (PMC10046465; doi:10.3390/biom13030421)
Supplement: Supplementary file 1 [file biomolecules-13-00421-s001.zip › Abbreviations.pdf]

Abbreviations

IC/BPS：Interstitial cystitis/bladder pain syndrome
HIC：IC/BPS patients with hunner's lesion
NHIC：IC/BPS patients without hunner's lesion
IIME：Inflammatory immune microenvironment
ICD：Immunogenic cell death
RCD：Regulated cell death
GEO：Gene Expression Omnibus
IRGs：ICD related genes
RF：Random forest
SVM：Support vector machine
ROC：Receiver operating characteristic curve
DCA：Decision curve analysis
IAMPs：ICD associated molecular patterns
KEGG：Kyoto Encyclopedia of Genes and Genomes
GO：Gene Ontology
ssGSEA：Single Sample Gene Set Enrichment Analysis
DEGs：Differentially expressed genes
WGCNA：Weighted correlation network analysis
PPI：Protein-protein interaction
PCA：Principal component analysis
qRT-PCR：Quantitative real-time polymerase chain reaction
FBS：Fetal bovine serum
