# Supplementary material for: Immunogenic Cell Death Associated Molecular Patterns and the Dual Role of IL17RA in Interstitial Cystitis/Bladder Pain Syndrome
Source: Biomolecules. 2023 Feb 23;13(3):421. doi: 10.3390/biom13030421 (PMC10046465; doi:10.3390/biom13030421)
Supplement: Supplementary file 1 [file biomolecules-13-00421-s001.zip › Table S1.pdf]

Supplementary table S1. Primer Sequences for q-PCR.

Species	Gene	Primer	Sequence（5'-3'）
Mouse	iNOS	F	CAAGCACCTTGGAAGAGGAG
Mouse	iNOS	R	AAGGCCAAACACAGCATACC
Mouse	CD32	F	AATCCTGCCGTTCCTACTGATC
Mouse	CD32	R	GTGTCACCGTGTCTTCCTTGAG
Mouse	CD16	F	TTTGGACACCCAGATGTTTCAG
Mouse	CD16	R	GTCTTCCTTGAGCACCTGGATC
Mouse	CD86	F	GACCGTTGTGTGTGTTCTGG
Mouse	CD86	R	GATGAGCAGCATCACAAGGA
Mouse	CD11b	F	CCAAGACGATCTCAGCATCA
Mouse	CD11b	R	TTCTGGCTTGCTGAATCCTT
Mouse	CD206	F	CAAGGAAGGTTGGCATTTGT
Mouse	CD206	R	CCTTTCAGTCCTTTGCAAGC
Mouse	Arg1	F	TCACCTGAGCTTTGATGTCG
Mouse	Arg1	R	CTGAAAGGAGCCCTGTCTTG
Mouse	IL-10	F	CCAAGCCTTATCGGAAATGA
Mouse	IL-10	R	TTTTCACAGGGGAGAAATCG
Mouse	CCL-22	F	CTGATGCAGGTCCCTATGGT
Mouse	CCL-22	R	GCAGGATTTTGAGGTCCAGA
Mouse	TGF-β	F	TGCGCTTGCAGAGATTAAAA
Mouse	TGF-β	R	CGTCAAAAGACAGCCACTCA
Mouse	Ym1/2	F	CAGGGTAATGAGTGGGTTGG
Mouse	Ym1/2	R	CACGGCACCTCCTAAATTGT
Mouse	IL12A	F	GCCAGGGTCATTCCAGTCTC
Mouse	IL12A	R	TTTCTCTGGCCGTCTTCACC
Mouse	IL12B	F	CTGCTGCTCCACAAGAAGGA
Mouse	IL12B	R	CAGGGGAACTGCTACTGCTC
Mouse	β-actin	F	GCACTCTTCCAGCCTTCCTTCC
Mouse	β-actin	R	GAGCCGCCGATCCACACG
Human	TNFα	F	ACCTCTCTCTAATCAGCCCTCT
Human	TNFα	R	GGGTTTGCTACAACATGGGCTA
Human	IL6	F	ACTCACCTCTTCAGAACGAATTG
Human	IL6	R	CCATCTTTGGAAGGTTCAGGTTG
Human	GSDMD	F	GGACAGGCAAAGATCGCAG
Human	GSDMD	R	CACTCAGCGAGTACACATTCATT
Human	IL10	F	GACTTTAAGGGTTACCTGGGTTG
Human	IL10	R	TCACATGCGCCTTGATGTCTG
Human	IL17RA	F	GACACTCCGCGACTGTTTC
Human	IL17RA	R	GCCCGTGATGAACCAGTACAC
Human	IL18	F	TCTTCATTGACCAAGGAAATCGG
Human	IL18	R	TCCGGGGTGCATTATCTCTAC
Human	IL1β	F	AGCTACGAATCTCCGACCAC

Human	IL1β	R	CGTTATCCCATGTGTCGAAGAA
Human	NFKB1	F	GAAGCACGAATGACAGAGGC
Human	NFKB1	R	GCTTGGCGGATTAGCTCTTTT
Human	NLRP3	F	CCACAAGATCGTGAGAAAACCC
Human	NLRP3	R	CGGTCCTATGTGCTCGTCA
Human	TLR4	F	AGACCTGTCCCTGAACCCTAT
Human	TLR4	R	CGATGGACTTCTAAACCAGCCA
Human	E-Cadherin	F	AAAGGCCCATTTCCTAAAAACCT
Human	E-Cadherin	R	TGCGTTCTCTATCCAGAGGCT
Human	TJP1	F	CTGGTGAAATCCCGGAAAAATGA
Human	TJP1	R	TTGCTGCCAAACTATCTTGTGA
Human	ICAM1	F	GTATGAACTGAGCAATGTGCAAG
Human	ICAM1	R	GTTCCACCCGTTCTGGAGTC
Human	BMP4	F	TAGCAAGAGTGCCGTCATTCC
Human	BMP4	R	GCGCTCAGGATACTCAAGACC
Human	GLI1	F	AACGCTATACAGATCCTAGCTCG
Human	GLI1	R	GTGCCGTTTGGTCACATGG
Human	β-actin	F	CTACCTTCAACTCCATCA
Human	β-actin	R	GAGCAATGATCTTGATCTTC
