# Supplementary figures and images for: Immunogenic Cell Death Associated Molecular Patterns and the Dual Role of IL17RA in Interstitial Cystitis/Bladder Pain Syndrome
Source: Biomolecules. 2023 Feb 23;13(3):421. doi: 10.3390/biom13030421 (PMC10046465; doi:10.3390/biom13030421)

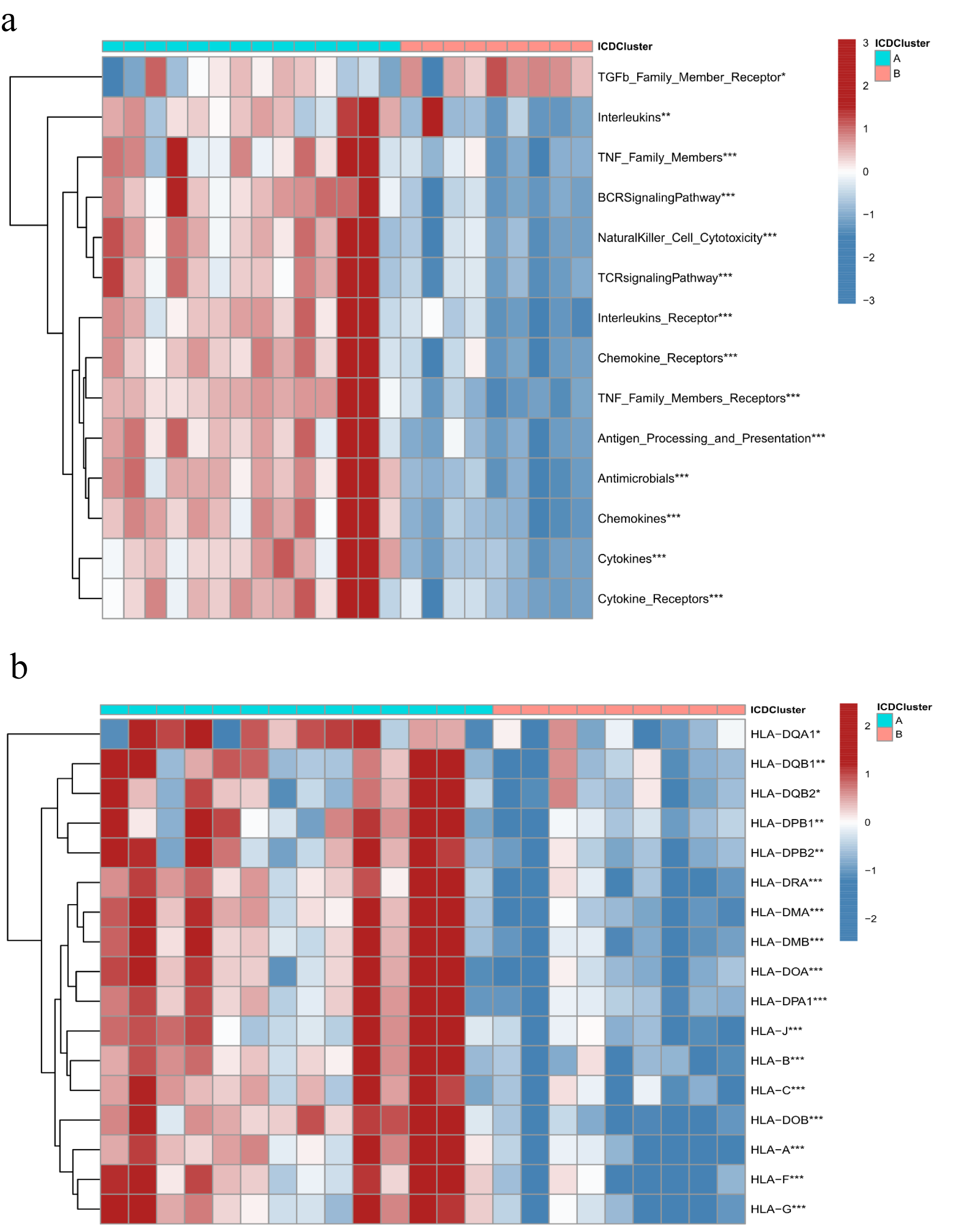

Supplement: Supplementary file 1 [file biomolecules-13-00421-s001.zip › Figure S1.tif]
